# Analyzing protein topology based on Laguerre tessellation of a pore-traversing water network

**DOI:** 10.1038/s41598-018-31422-5

**Published:** 2018-09-10

**Authors:** Jérémy Esque, Mark S. P. Sansom, Marc Baaden, Christophe Oguey

**Affiliations:** 1 LPTM, CNRS UMR 8089, Université de Cergy-Pontoise, 95302 Cergy-Pontoise, France; 2 LISBP, Université de Toulouse, CNRS, INSA, INRA, 135 Avenue de Rangueil, 31400 Toulouse, France; 3 Department of Biochemistry, University of Oxford, South Parks Road, Oxford, OX1 3QU United Kingdom; 4 Laboratoire de Biochimie Théorique, CNRS, UPR9080, Univ Paris Diderot, Sorbonne Paris Cité, 13 rue Pierre et Marie Curie, 75005 Paris, France

## Abstract

Given the tight relation between protein structure and function, we present a set of methods to analyze protein topology, implemented in the VLDP program, relying on Laguerre space partitions built from series of molecular dynamics snapshots. The Laguerre partition specifies inter-atomic contacts, formalized in graphs. The deduced properties are the existence and count of water aggregates, possible passage ways and constrictions, the structure, connectivity, stability and depth of the water network. As a test-case, the membrane protein FepA is investigated in its full environment, yielding a more precise description of the protein surface. Inside FepA, the solvent splits into isolated clusters and an intricate network connecting both sides of the lipid bilayer. The network is dynamic, connections set on and off, occasionally substantially relocating traversing paths. Subtle differences are detected between two forms of FepA, ligand-free and complexed with its natural iron carrier, the enterobactin. The complexed form has more constricted and more centered openings in the upper part whereas, in the lower part, constriction is released: two main channels between the plug and barrel lead directly to the periplasm. Reliability, precision and the variety of topological features are the main interest of the method.

## Introduction

To carry out their function, proteins form many intra-molecular interactions but also important interactions with the solvent^1^, and with the lipid bilayer in the case of membrane proteins. Topology is a prominent aspect of both morphology and functioning of proteins. The topology-function relationship has been used to classify protein domains^2,3^.

In order to better analyse the topology of proteins, protein complexes and their environment, we propose a panel of methods based on rigorous mathematical grounds, implemented in the numerical program VLDP (Voronoi Laguerre Delaunay Protein)^4–6^, freely available as web server^6^ or upon request to the authors.

The first task performed by VLDP is to build the Laguerre, or weighted Voronoi, diagram of the data, typically a set of positions describing the molecular structure as provided by bio-structural techniques, e.g. crystallography, NMR spectroscopy or simulation (molecular dynamics, Monte-Carlo, etc.). Many such structures are registered in molecular data banks such as the PDB or NDB^7^. The Laguerre space partition is the basis underlying all the implemented topological analysis methods: inter-atomic, inter-residue or inter-molecular contacts, connected components of the bulk or of the surface, optimal paths, porosity, genus of the surface, stratification. VLDP computes elementary metric quantities such as volume, surface and polygonal interfacial area and was previously used in extensive statistical analyses of globular proteins^4,5^. As an alternative to previous approaches^8^, here we mostly demonstrate how to characterise the protein porosity, pockets, tunnels and paths, using VLDP.

**Figure 1:**
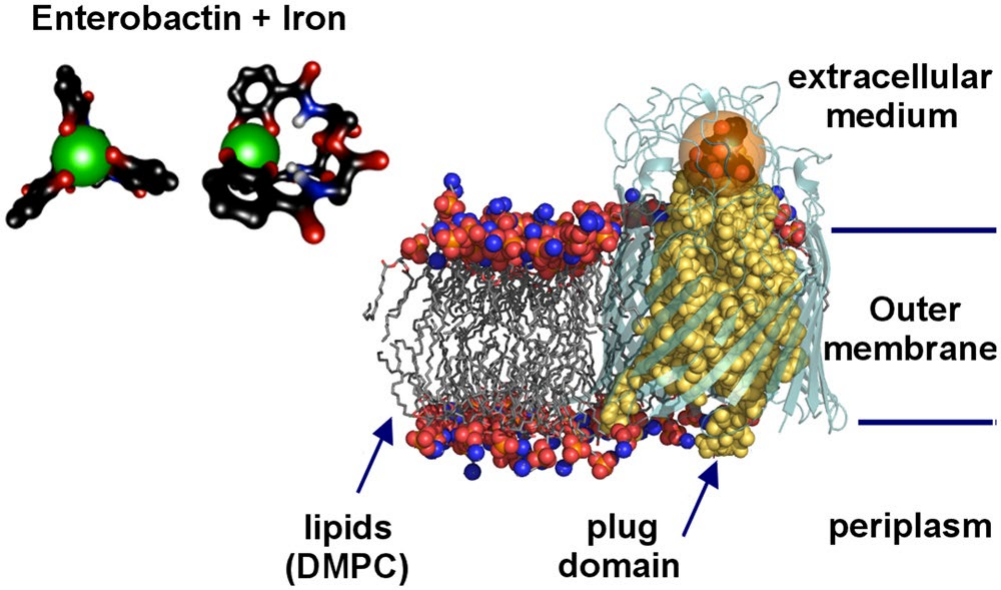
General view of the biological test-case. The FepA membrane protein is surrounded by a lipid bilayer separating the extracellular medium and the periplasm. The main part of FepA is represented in lightteal cartoon mode whereas its plug domain is in yellow balls. The lipids on the left are in balls and sticks. A transparent orange sphere of 8 Å radius shows the size of the enterobactin-iron complex at the right scale. The inset provides enlarged top and side views of the enterobactin iron chelator.

Membrane proteins, such as those operating transport through the membrane in a controlled way, are prone to show interesting topological properties related to their function. Our study uses classical algorithms such as space partitions and Dijkstra paths search^9^, applied in an original and novel way by taking into account the full representation of a membrane protein including the solvent and the lipid bilayer (Fig. 1). In conjunction with a suitably weighted Laguerre tessellation, this complete surrounding yields a more precise description of the interfaces, in particular of the protein surface^4,5^. In contrast to existing Voronoi-based programs mainly searching empty space within isolated structures^10–12^, VLDP incorporates physico-chemical effects related to hydration, ions, polarisation, lipids, etc.

The methods will be demonstrated on a test case: FepA, the ferric enterobactin permease. The FepA transporter is a siderophore binding protein in the outer membrane of *Escherichia coli* involved in feeding the bacteria with iron. The Fe3+ ion is first captured by enterobactin, a small siderophore produced by the bacteria; then the Fe-enterobactin complex binds to FepA and is transferred to the periplasmic side by a mechanism which is still not understood. FepA consists of three main parts^13^: a globular plug domain^14^ obstructing a *β*-barrel enclosed in the lipid bilayer, and a set of loops on the external side. The plug-domain is a specific feature of this transporter family, compared to other *β*-barrel transporters forming hollow pores like OmpA^15^.

Molecular dynamics simulations^16–20^ and computational analyses^21,22^ have been performed to gain insight into various aspects of iron transport. Here, VLDP is applied to FepA, both as a first application to a membrane protein and as means of investigating the often complex geometry related to the iron transport function. Of particular interest is the question of whether one can detect any change induced by the presence of the siderophore. That is why FepA was modelled by molecular dynamics both in absence and in presence of the ferric enterobactin. The system was simulated in conditions retaining the essential components of the real environment: a lipid bilayer and a solvent of ions and water molecules. The ligand-free form of FepA has been solved by crystallography^23^ and simulated by MD^18^, yet including the enterobactin is an additional original aspect of this study allowing us to probe the sensitivity of the method in detecting changes in water networks. This study endeavours to qualitatively and quantitatively characterise water pathways of possible functional importance in FepA. To ensure its functioning, the protein structure should have a topology appropriate to uptake and transport the iron-enterobactin complex. The methods shown in this paper can be used in a wider range of applications.

## Results

### Connected components: Water passage and water pockets

Regarding trans-membrane proteins, one of the first questions is the existence of water channels connecting the extra-cellular medium and the interior, or periplasm in double membraned cells. An answer is provided by the connected components of the water medium. Space on both sides of the membrane is filled with water molecules. Some water molecules are also localised inside the protein, either trapped or forming channels connected to the external media. For FepA, in all but one molecular dynamics (MD) snapshots, in presence and in absence of ligand, there is a single main connected component; therefore at least one water path links the extracellular side to the periplasmic side. The one and only exception found is the complexed form snapshot at 82 ns, which does not contain any water path completely crossing the protein; therefore the two water domains on each side of protein and membrane are disconnected in this case.

**Figure 2:**
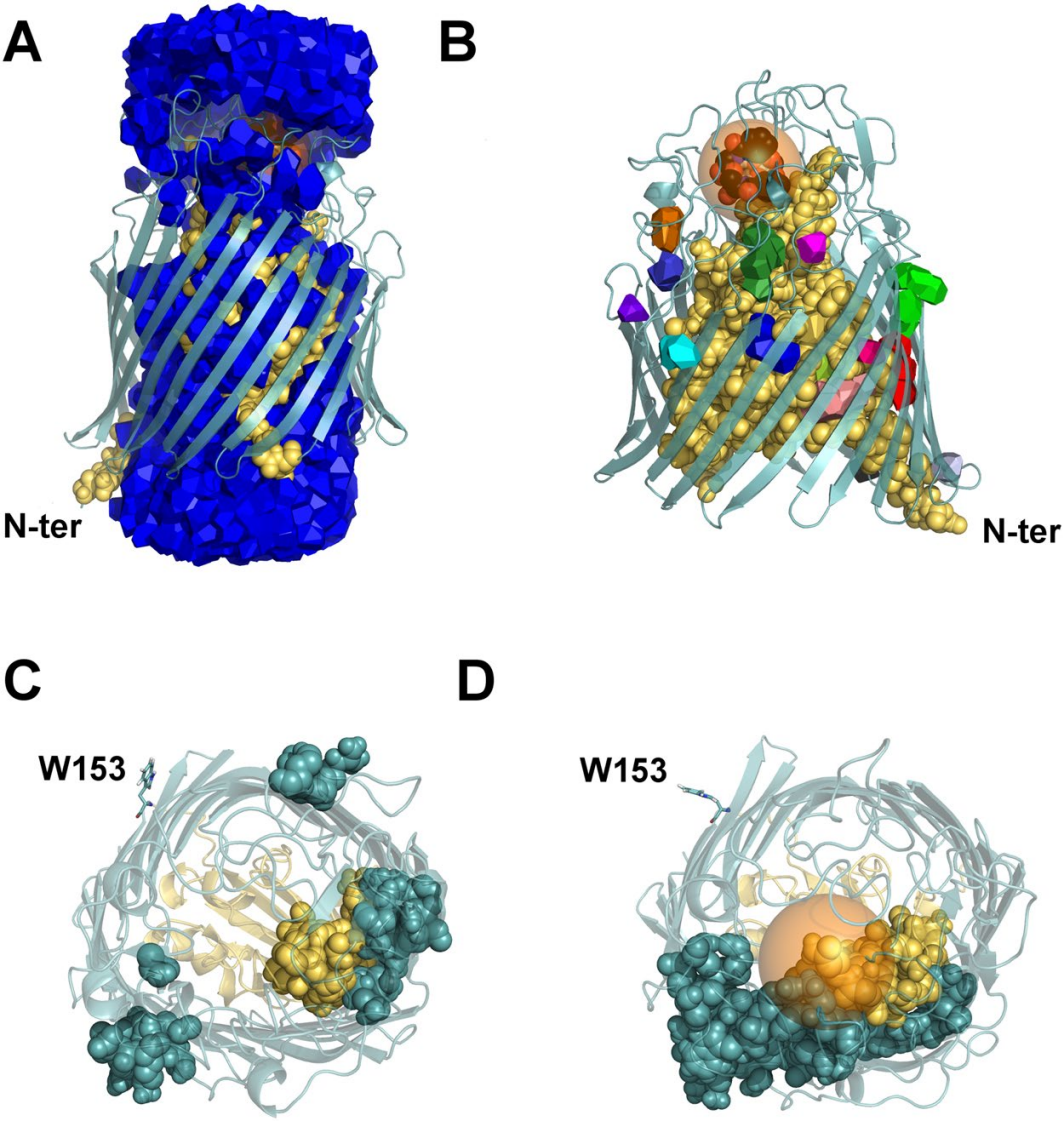
Localisation of connected components of water in FepA. (**A**) Main connected component of water. A water path through the protein bridges the media on both sides of the membrane, whence the main connected component, in blue, extends on both sides of the membrane. (**B**) Isolated water clusters, each painted a different colour (for visual convenience). The FepA protein (at 85 ns) is displayed in cartoon mode for the peripheral part, in yellow Van der Waals balls for the plug, while the water molecules appear as Laguerre polyhedra. The semi-transparent sphere of 8 Å radius highlights the enterobactin. (**C** and **D**) Show the spatial distributions of water inclusions in a top view of typical snapshots of the ligand-free (70 ns) and complexed (85 ns) forms respectively. The van der Waals balls represent the residues permanently bordering water pockets. The transparent orange sphere indicates the position of the enterobactin, not shown in view D. As reference points, N-ter (A and B) and tryptophan W153 (C and D) are labelled. W153, rendered in sticks, marks the end of the plug domain and the beginning of the *β*-barrel.

Beside the main components, the algorithm finds a number of smaller connected components, whose statistics are summarised in Table S1 for the MD snapshots of FepA in both ligand-free and complexed forms. The main components are not included in the counts. The other, smaller, components are either in the protein interior, or squeezed between the protein and the lipid bilayer, forming inclusions of isolated or aggregated water molecules. Figure 2A,B display, respectively, the water main component and the inclusions in one snapshot of FepA. The count of water molecules — the population — in each cluster ranges from 1 to 16 with a maximum occurring in the snapshots at 40 and 45 ns without ligand. In the final period of the MD simulation, we found on average *N*_inc_ = 12.5 inclusions of (mean ± rms) population 1.9 ± 1.7 for FepA ligand-free; the values for FepA complexed with enterobactin were *N*_inc_ = 14.6, pop = 2.7 ± 2.1. On average, the number and size of water pockets are marginally higher in the complexed form. See Table S1 and Fig. S1 for detailed distributions. However, if we consider the iron-enterobactin complex as part of the solvent, on average two water pockets disappear from the count (see Fig. S2). This is because the enterobactin is always (at least in the analysed snapshots) in contact with the main water connected component (network), as we will see in the section on widest paths.

Another difference between the ligand-free and complexed forms of FepA is the mobility of water pockets, as detected by listing the residues in contact with these pockets (see Fig. 2C,D). Whereas in the ligand-free form water pockets migrate relative to the enclosing residues, in presence of the enterobactin the permanently contacting residues tend to gather together in one half of the protein (the lower part of Fig. 2D). These water inclusions appear more stable in the presence of enterobactin. This observation implies that in the other half, indicated by residue W153 in Fig. 2D, the water present is mostly involved in the main connected component, that is, in the water channel network connected to the outside of the protein (depicted in blue in Fig. 2A).

### Disjoint paths: Bottlenecks and channel constrictions

Connected components informed us that, most of the time, at least one path goes all the way through the protein. But there may be several crossing water paths. So, the disjoint path search algorithm was used to probe the porosity of the protein and channel constrictions.

Table 1 summarises the counting of disjoint paths for both FepA forms: complexed or not. In all snapshots, the number of disjoint paths remains low, between 1 and 3, except for a few early snapshots depending on the MD initial conditions. The small number of disjoint paths indicates the presence of constrictions in the water network, where the paths are somehow forced to overlap. Most often, these constrictions are either on the top of the protein (extracellular side) or near FepA’s barrel centre. As expected, this limitation is due to the plug forming an occlusion for water molecules, but also to the ligand present in the complex.

**Table 1:** Count of disjoint water paths in the successive snapshots.

ligand-free FepA				FepA + enterobactin			
time (ns)	DP	e-chan.	p-chan.	time (ns)	DP	e-chan.	p-chan.
0	6	3	1	0	5	2	1
5	4	2	1	5	5	3	1
10	2	1	1	10	4	4	1
15	4	3	1	15	3	3	1
20	4	3	1	20	4	4	1
25	2	2	1	25	2	2	1
30	2	2	1	30	2	2	1
35	2	1	1	35	3	3	1
40	2	2	1	40	3	3	1
45	2	2	1	45	3	3	1
50	3	3	1	50	2	2	1
55	2	1	1	55	2	2	1
*60*	1	1	1	60	2	2	1
*61*	1	1	1	65	2	2	1
*62*	3	3	1	70	2	2	1
*63*	2	2	1	75	2	2	1
*64*	2	2	1	*76*	2	2	1
*65*	2	1	1	*77*	2	2	1
*66*	2	2	1	*78*	1	1	1
*67*	3	3	1	*79*	2	2	1
*68*	1	1	1	*80*	2	2	1
*69*	2	2	1	*81*	1	1	1
*70*	3	3	1	*82*	0	0	0
				*83*	2	2	1
				*84*	2	2	1
				*85*	2	2	1
				*86*	2	2	1
*avg (60–70)*	2.00 ± 0.77			*avg (76–86)*	1.64 ± 0.67		

The columns give respectively the number of disjoint paths (DP), the number of openings through which they enter the protein from the extracellular domain (e-chan) and the number of openings through which they get out to the periplasm (p-chan). The bottom line indicates the average (±rms) value of DP over the last 10 ns.

The count of openings in Table 1 shows that FepA, and hence the water network through it, is not symmetric. On the periplasmic side, all the paths get out through FepA’s flared conical opening (p-chan = 1). On the extracellular side, FepA has many more narrow entries. Indeed, the entry region is cluttered by extracellular loops of the protein. Moreover, branching occurs in the core of the protein obstructed by the plug domain. Water aggregates into a number of scattered small channels.

**Figure 3:**
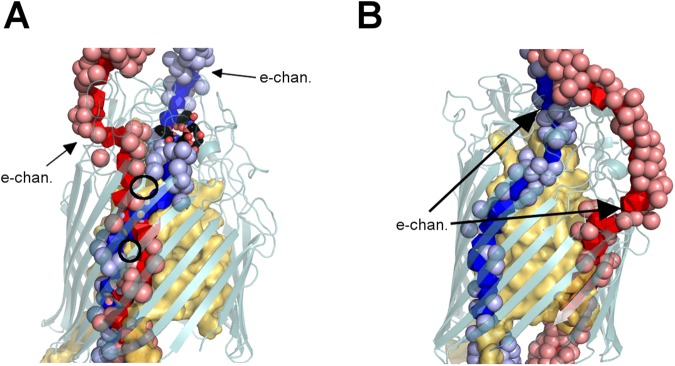
Two configurations of disjoint water paths through FepA. Disjoint shortest water paths are displayed in blue/red in (**A**) the complexed form at 85 ns, (**B**) the ligand-free form at 69 ns. In (**A**), the two disjoint paths share common channel portions around the plug, whereas in (**B**) the two disjoint paths follow well separated channels. The black circles in (**A**) point out spots where the paths are locally separated. Labels “e-chan” indicate openings listed in Table 1. In the complexed form (**A**), the enterobactin is shown in sticks. The balls represent the first layer of water (Oxygen atoms) around the disjoint paths.

By direct observation, also partly indicated in Table 1, we see that nearly all possible combinations occur in FepA’s different snapshots. In the case when the number of disjoint paths equals 1, only one water path crosses the protein and any other tentative path gets stuck at constrictions (narrow passages) or dead ends. When the count of disjoint paths exceeds 1, either several paths group together to form a single, wider channel, possibly diverging further away or not (see Fig. 3A); or the paths remain separated in parallel channels all along through the protein (see Fig. 3B).

The stability of the listed paths was examined by changing the Laguerre weight of water (see Fig. S3 and Table S2). Changing weights causes contact switches and flips of Delaunay edges, creating or disrupting links between water molecules. Increasing weight increases the volume of the corresponding Laguerre polyhedra and tends to set new contacts between water molecules. Conversely, decreasing the water weight tends to break some contacts defining paths. The consequence is the appearance or disappearance of several paths as shown in Table S2. The general tendency is that the disjoint paths are fairly stable for reasonable weight variations. Down to 0 weight, a few paths disconnect indicating that the channels are narrow in places.

This parametric stability is meant to indicate a correlated stability in time, not directly probed in the present study.

### Genus of the surface: porosity of the protein

A global indication of the porosity of the protein is given by the genus *g* of its surface. Essentially, *g* counts the number of pores. In the algorithm, the genus is computed from the tessellation using Euler’s equation, Eq. (2) in *Methods*.

**Figure 4:**
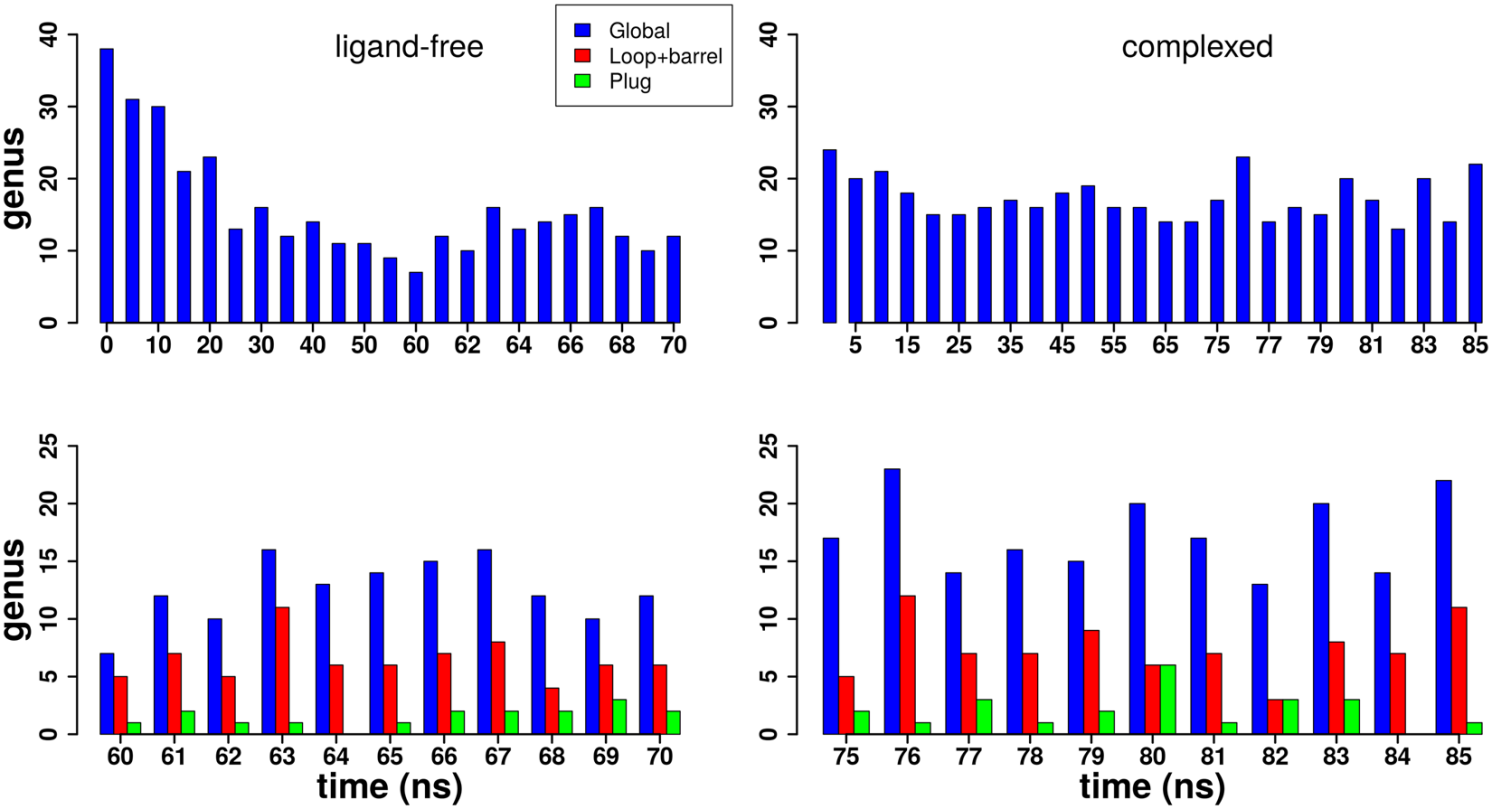
Genus *g* of the FepA surface as function of MD time. The bar plot time series show FepA’s genus in the ligand-free form (on the left) and in the complexed form (on the right). Top: only the genus *g* of the whole protein surface is displayed. Bottom: the surface genus of the *β*-barrel and plug are also given during the last ten ns.

Figure 4 displays the genus *g* of three parts of the protein at the sampled MD times: the entire protein, the peripheral part and the plug-domain. First of all, specially for the ligand-free form of FepA, note a slow transient decrease of the global genus over the first tens of nanoseconds. This higher porosity during the equilibration was already noticed when we analysed disjoint paths. So, thermodynamic equilibrium sets up only in the second half of the simulation. The global genus appears as a good indicator of some slow relaxation modes.

Figure 4 also shows that the global genus is bigger in presence of the enterobactin ligand, FepA’s porosity is higher. Indeed, the mean global genus over the last 10 ns is about 12.5 ± 2.8 for the ligand-free form versus 17.3 ± 3.4 for the complexed form.

Given the possibly ambivalent status of the ligand, we also computed the surface genus of the union of the enterobactin-iron complex and the protein, taken as a single body. Compared to the protein alone, displayed in Fig. 4, the united genus *g* is less, by 1 to 3 units on average. Some holes are shut by the internal ligand (−1), but others are subdivided into finer passages (+1 for each new route) (see Fig. S4); the balance is negative.

**Figure 5:**
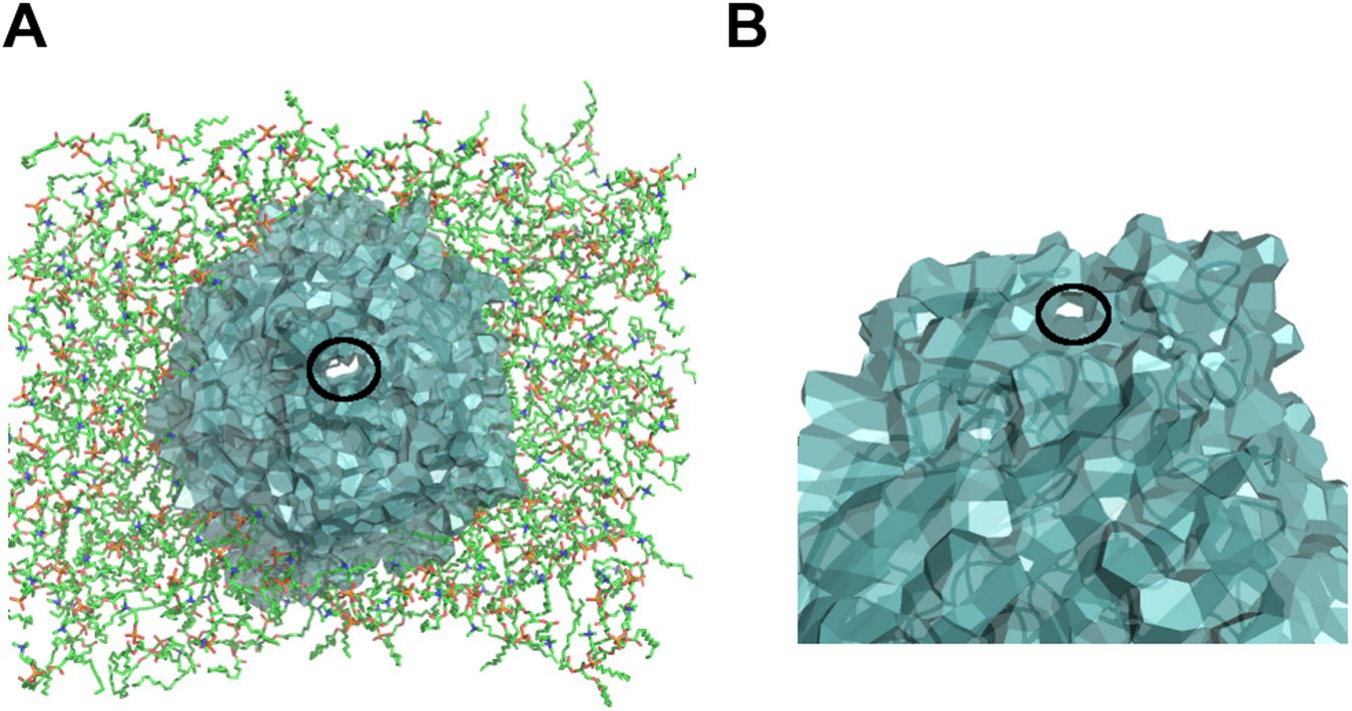
Contributions to the peripheral genus. (**A**) Axial channel crossing the entire protein. (**B**) Peripheral passage, or handle, formed by protein loop contacts in the extracellular domain. In both cases, the hole contributing one unit to the genus is encircled in black. The protein is shown in cartoon mode and Laguerre surface; the lipids in (**A**) are shown in sticks. The structure was taken at 81 ns of the complexed form MD.

However, as a topological number, the genus *g* counts all the water passages without any respect to their size, position, or direction. Contributions to the global genus *g* include channels crossing the protein (see Fig. 5A) as well as “handles” that start and end on the same side of the protein (mostly the extracellular side) (see Fig. 5B). Indeed, FepA has several more or less flexible loops on the exterior side, as illustrated in Fig. 5. Moreover, passage is tightly controlled by the plug domain.

Most applications, in particular transport, will require additional information on, *e*.*g*., the size, length or localisation of the pores. As a first step towards sorting out the contributions to the genus, additional calculations were performed on the last 10 ns by subdividing the protein into two parts: (*i*) the peripheral part, comprising the loops and the *β*-barrel; (*ii*) the plug domain only. In all cases (see Fig. 4 bottom), the plug has low genus, confirming its globular nature. The low genus of the plug domain comes from small “handles“ on its surface. For the peripheral part, the porosity comes from the hollow channel in the barrel, counting for at least one unit in the genus, and entries or loops in the extracellular part as shown on Fig. 5. However, note that the global genus is not the sum of the partial genera (peripheral and plug domain). Roughly speaking, the discrepancy counts the channels specifically due to the plug-periphery association. It is a balance between channels that do not exist, or exist only, when both parts are taken separately.

For instance, looking at the snapshots 64 ns of the ligand-free form and 84 ns of the complexed form, we see that the genus of the plug domain is equal to 0 but the global genus and the peripheral genus are not equal. The periphery alone has a low genus, below 8. The global genus is twice larger. The difference counts the pores sandwiched between the plug and the periphery. Indeed many narrow channels around the plug replace the single wide passage through the empty barrel.

The high value of the genus is an indication of the complexity of the water network. The larger value, in the complexed form of FepA, of *g* and of the difference between the global and partial genus, are first indications of some loosening of the contacts between the plug and peripheral parts caused by internal hydration.

### Widest paths enumerate the transport possibilities

So far, only topology has been used. In this section, length and distance are measured to determine the widest water channels through the protein.

The algorithm finds the least costly paths for a cost function penalising short distance with any protein atom, given by Eq. (3) in *Methods*. The paths are constrained to visit water positions exclusively. This is a major difference with other Voronoi-based methods such as MOLE^10^. Comparison of the methods, solvent-based and isolated, is displayed in the supplements (Figs S5 and S6). Globally, both methods are similar. However in a few cases, the MOLE-like method yields optimal paths through the protein that do not completely follow water pathways. In places, the small water molecules are squeezed in channels thinner than empty interstices between residues, presumably hydrophobic. That is why we chose paths following water molecules. In principle, our solvent-based method yields a channel radius equal to or smaller than the locally optimal axis (from geometric MOLE-like methods), but in practice the effective radius discrepancy is small (see Fig. S6), negligible compared to the benefit of the hydrophilic environment.

All the paths investigated in this section were constrained to start at the enterobactin binding site and end in the periplasm. Searching several disjoint optimal paths by iterating the algorithm with strict mutual exclusion (*d*_*p*_ = 0.1, see paragraph *Widest Paths* in section *Graph Theory Methods*), leads to a very limited number of disjoint paths, never more than three in the last 10 ns of both simulations of FepA. This range is similar to that of crossing shortest paths found previously.

**Figure 6:**
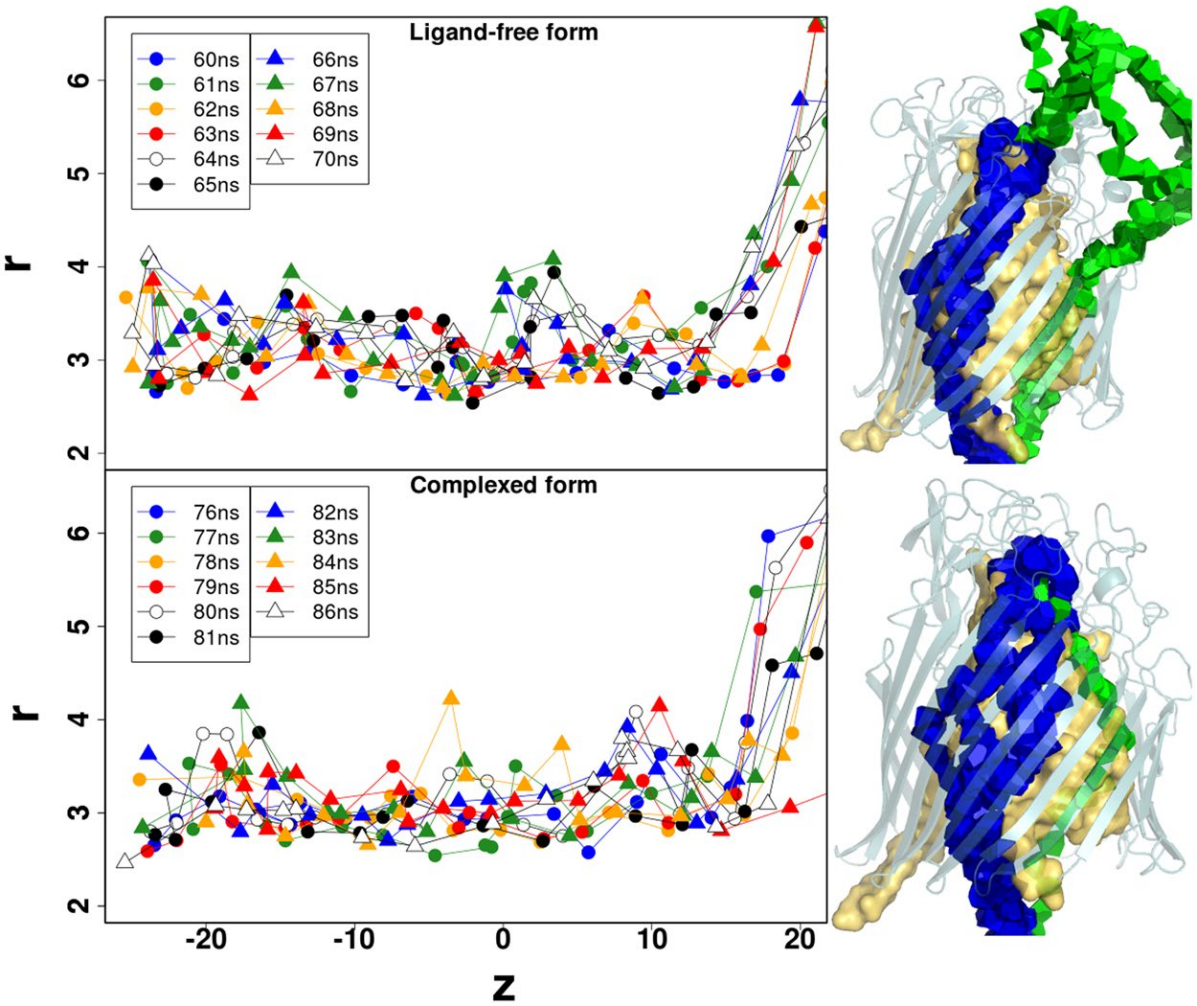
Optimal channel radius as a function of ***z***. On the left, the radius profiles of the widest direct paths are plotted for all snapshots in the last 10 ns of the ligand-free form (top) and complexed form (bottom) simulations of FepA. The origin *z* = 0 is at the protein centre. On the right is shown the Laguerre representation of all the widest direct paths used for the radius plot. The paths are coloured according to their localisation around the plug domain. The blue direct paths are conserved in both forms; whereas green paths appear as indirect in the ligand-free form (top right) and potential precursors of direct paths in the complexed form (bottom right).

A peculiar effect of the cost function (3) is the appearance of *indirect* optimal paths, that first escape back into the external domain, where cost is low, and cross the protein through another opening. Because the goal is to analyse possible water passages from the enterobactin binding site, these indirect paths are not taken into account. So, to quantify the width of possible passages, we plotted the radius of the widest *direct* paths in the last 10 ns of the simulations (see Fig. 6). Direct paths connect the enterobactin site to the periplasm directly through the protein barrel. No significant difference between the ligand-free and complexed forms of FepA can be drawn from these plots. Indeed, the average radius is 3.2 ± 0.5 Å in both cases. As this radius measures the distance to the surrounding atomic centres, a van der Waals radius of the order of 1.1 Å should be subtracted, yielding an effective channel radius of 2.1 ± 0.5 Å.

More interesting is the occurrence of a wide path (green), direct in the presence of the ligand, that matches the internal part of an indirect path in the ligand-free form (see Fig. 6 and Table S3). The widest path is always direct in the complex dynamics. Indirect paths rank first, with the lowest cost, only in a few snapshots of the ligand-free form of FepA.

**Figure 7:**
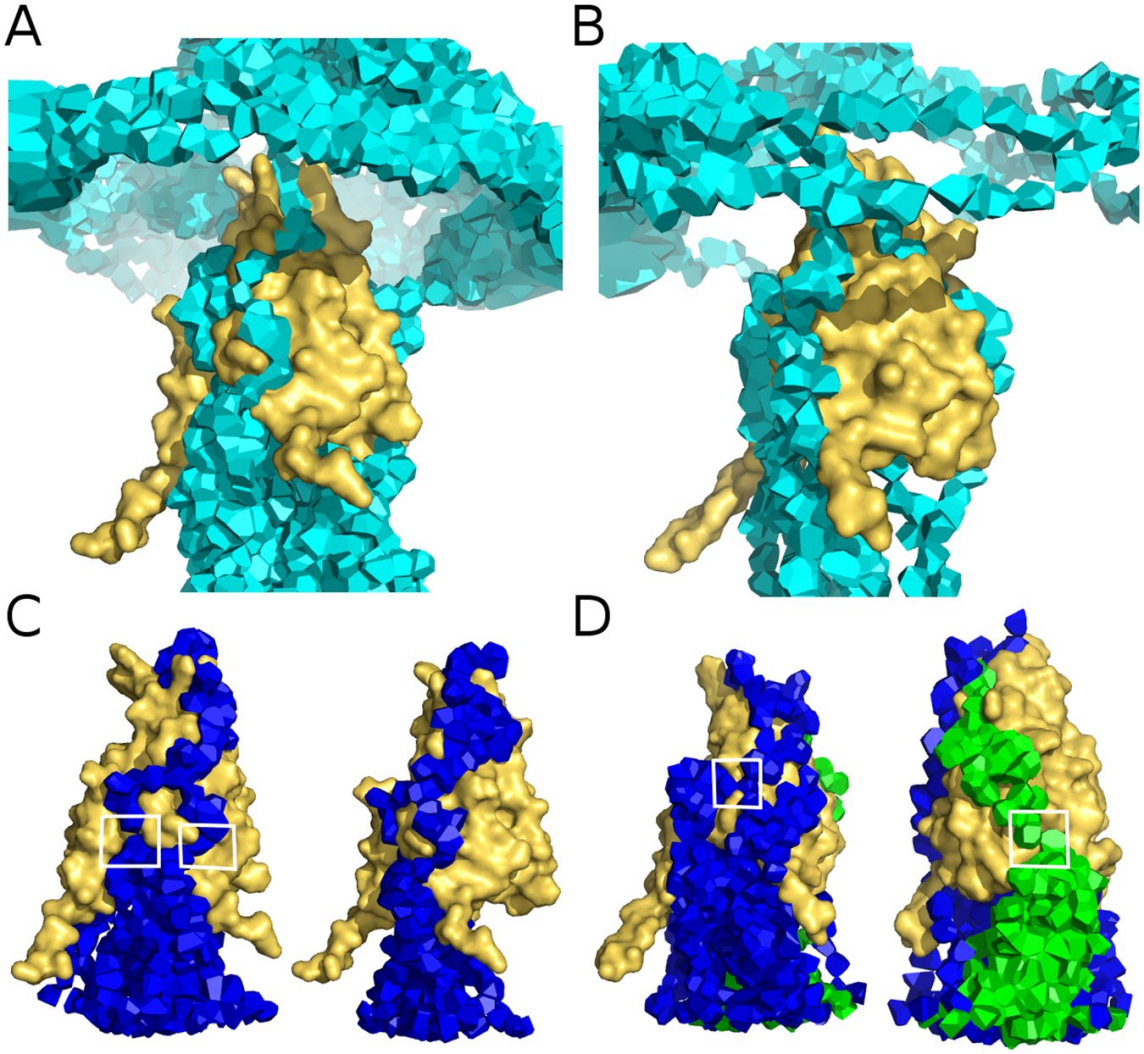
Widest water channels through FepA. Some widest paths found with a soft penalty are displayed for the ligand-free (70 ns, (**A**,**C**)) and complexed (86 ns, (**B**,**D**)) forms of FepA. On the top, only the indirect paths are displayed whereas the bottom shows the direct paths for both forms with the same colour code as in Fig. 6. The white squares localise bottlenecks. The plug domain is shown in yellow surface.

To get a better sampling of the water network, the strict exclusion was changed to a softer condition accepting small overlaps with a penalty (*d*_*p*_ = 1 in paragraph *Widest Paths* of Sec. *Graph Theory Methods*). By iterating the search, more optimal paths accumulate, tracing out wider channel portions and revealing the location of bottlenecks (places where most paths overlap) (see Fig. 7).

In FepA without ligand, the direct paths are squeezed into a limited number of sparse channels (maximum two). The indirect paths are quite numerous and find a way through a number of openings located on the protein periphery. In the presence of enterobactin, particularly in the last snapshots, the internal water network appears more spread out and branched. Moreover the indirect paths are less numerous, implying a slightly more hermetic periphery.

These features reveal subtle changes in the channel network, compatible with a lateral constriction in the upper part of the protein, where the enterobactin is inserted, significantly reducing the permeability at this level but slightly enhancing the porosity towards the periplasm in the lower part.

### Changes in the plug domain in the presence of enterobactin

Because of the important, but yet unsolved, role attributed to the plug-domain in the porosity and topology of the transporter, detailed measurements were performed on the interaction of the plug domain with its surrounding over the last 10 ns of the simulations. First, the volume of the plug domain was evaluated by summing the volume of its residues. Second, the effect of the presence of enterobactin was probed by examining the variations of the plug domain contacts.

On average, the plug domain volume decreases by 0.8% in presence of enterobactin: from 17422 ± 97 Å^3^ without ligand to 17287 ± 87 Å^3^ with ligand. The difference of about 135 Å^3^ is just above the standard deviations. An analysis by residue shows that Arginine 132 (R132) is strongly influenced by enterobactin: ${\overline{V}}_{{\rm{free}}}-{\overline{V}}_{{\rm{comp}}}$ is about 18 Å^3^. The movement of its side chain is reduced by a strong interaction with the backbone in the ligand-free form; whereas it is more flexible and extended in the complexed form. The presence of more water may explain the facilitated residue mobility observed for R132 in the presence of the enterobactin.

**Figure 8:**
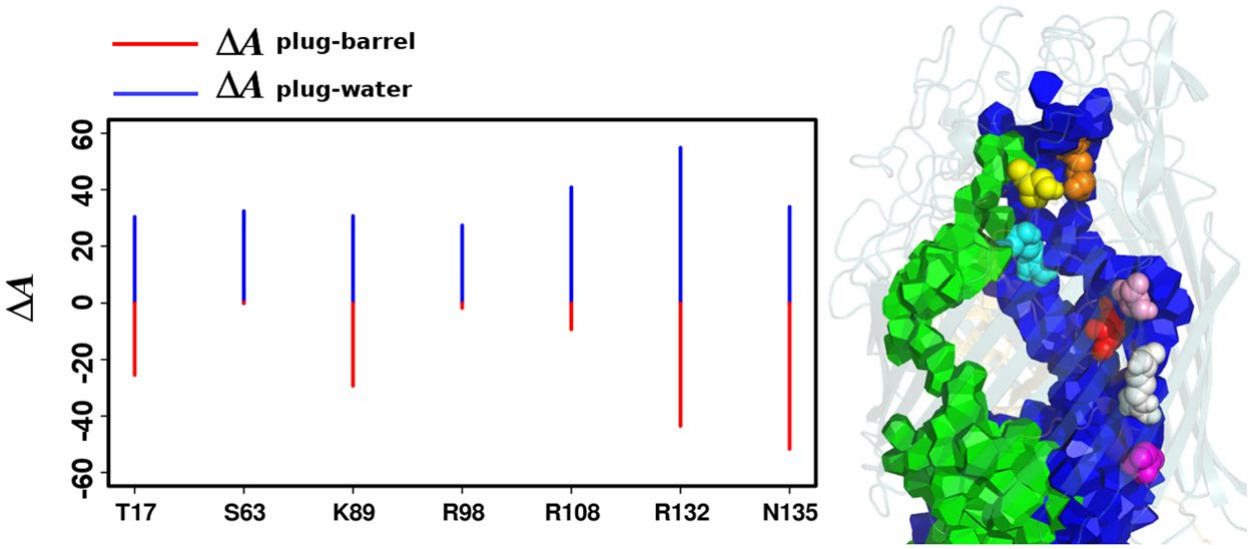
Key residues and plug contact differences between ligand-free and complexed forms. The key residues were selected by the following criteria on the change Δ*A* = *A*_comp_ − *A*_free_ in contact area: Δ*A*(plug − barrel) < −20 Å^2^ or Δ*A*(plug − water) > 20 Å^2^. The residues are listed horizontally and labelled as in the original PDB file (on the left). On the right, the FepA structure at 86 ns with the ligand is shown in transparent cartoon. The two main water channels (blue and green) are displayed in Laguerre polyhedra. The key residues are shown in spheres: T17 (magenta), S63 (yellow), K89 (red), R98 (orange), R108 (cyan), R132 (white), N135 (pink).

As a complementary approach, the contact area of the plug with its surrounding was calculated and reported in Table 2. A residual analysis identifies some key residues primarily involved in the plug-barrel contact loss: T17, S63, K89, R98, R108, R132, N135 (see Fig. 8). The dominant feature is a significant decrease of the contacts with the rest of the protein, the periphery (−3.2%). This decrease can be decomposed into three contributions: the contacts with enterobactin (0.8%), wetting (0.3%), and a global smoothing of the plug surface (2.1%). Indeed, according to Table 2, the total surface of the plug decreases by Δ*A*_p_/*A*_p_ = −2.1%, a little more than what a simple compression without shape modification would predict: Δ*A*_p_/*A*_p_ = (2/3)Δ*V*/*V* = −0.5%. Note that the contacts with water, which barely change in absolute value, become significantly larger in relative value: Δ*A*(plug − water)/*A*_p_ = 0.3% is 2.4% above the total area variation, just beyond the fluctuations *σ*(*A*_p_)/*A*_p_ = 1.3%. Once shape change is taken into account, the plug is wetter in the presence of the enterobactin than in its absence. The plug shrinks to a slightly more compact and less corrugated morphology via contact losses with the periphery but without reduction of its wet surface. The net result of the presence of the ligand is a slight detachment of the plug from the periphery, coupled to some spread of the water channels. This release is also indicated by the internal radius of the bottom part of the barrel (towards periplasm), larger by 7% in the complexed form than in the ligand-free form (see Fig. S7); and also shown by the moments of inertia (see Fig. S8). The plug itself does not swell up. Its volume slightly decreases and its quadratic moments show a small shortening along the vertical axis (see Fig. S8). The fact that the water channel radius does not increase (see Fig. 6) can also be understood as concurrent channel spreading and flattening.

**Table 2:** Contact area of the plug domain with surrounding.

contacts	ligand-free		complexed		difference	
	*A*(Å^2^)	*σ*(Å^2^)	*A*(Å^2^)	*σ*(Å^2^)	Δ*A*(Å^2^)	Δ*A*/*A*_p_
plug – *β* barrel	2597	57	2481	58	−116	−1.4%
plug – periphery	3175	68	2915	79	−260	−3.2%
plug – enterobactin	—	—	65	5	65	0.8%
plug – water	5100	105	5126	83	26	0.3%
total plug surface *A*_p_	8275	93	8106	81	−169	−2.1%

The contact area *A* is the area of interface, plug-(periphery/barrel/ligand/water), averaged over the last 10 ns; *σ* is the corresponding standard deviation. Δ*A* = *A*_comp_ − *A*_free_ is the difference between the complexed and ligand-free forms.

## Discussion

Hydration is fundamental for proteins, and a common approach, amongst a wide list of methods, focuses on anomalous diffusion of water near the protein, hydration sites, residence time, etc.^24–26^. The present study takes a complementary point of view, more oriented towards the solutes. It yields a detailed description of the pores, water channels in the protein, and of their evolution. Attention is payed, not to the individual molecule motion, but to the protein-solvent interface as a complete, complex and evolving surface. To do so, the VLDP software was used to analyse a series of MD snapshots from a fully solvated system. Compared to other grid or Voronoi based algorithms defining pores as empty places in the protein structure (CAVER, HOLE, MOLE, etc.), our method finds water-filled channels and pockets, which is an advantage for soluble molecules like enterobactin, sensitive to the physico-chemical properties, e.g. hydrophilicity, of confined passages.

The FepA membrane protein was chosen as a test case due to its topological intricacy. Compared with other *β*-barrel porins, as the Outer Membrane Protein (OMP) family, FepA has a N-terminal plug-domain (148 residues) folding up inside the barrel and completely occluding the barrel’s interior. This topology appears incompatible with the main biological function: the iron-enterobactin (circum-radius 8 Å) transport through the protein. So, FepA in both forms, ligand-free and complexed with enterobactin, was simulated by MD in a complete environment including the lipid bilayer and solvent, to investigate spontaneous rearrangements due to the presence of the ligand. Using VLDP, the topological analysis shows that FepA contains a significant amount of water distributed among connected channels and separated inclusions. This picture is dynamic; contacts between water components set on and off due to local agitation rather than long distance displacements. The channel network is composed of several openings in FepA’s extracellular surface, many branches and bottlenecks in the protein core, and a single wide conical opening towards the periplasmic region.

In the pool of investigated snapshots, all but one had a continuous water path bridging the extracellular and the periplasmic media. Often, several channels of fluctuating width and length exist. But the water paths are constrained by bottlenecks along traversing channels, as shown by the maximum number of three disjoint paths through FepA. This bound is stable in time, against perturbations and against the presence or not of the siderophore.

The differences between the ligand-free and complexed forms of FepA are quite subtle, but VLDP is precise enough to detect a few significant ones. A first difference is the mobility of water pockets, which are more gathered together in a specific region in the complexed form. A second difference between the two forms of FepA is the genus *g*, on average larger in the complexed form than in the ligand-free one. So, globally, the number of passages indicated by the genus is larger in the presence of the ligand. This trend remains within fluctuations.

The most noticeable changes are in the network topology. In absence of siderophore, trans-protein passages are either parallel or blocked, leaving only an exiguous passage from the enterobactin site to the periplasm. In presence of enterobactin and in the last stage of the MD, two main channels settle down durably. One of them is wider, and the other shares a common part with a hypothetically precursory indirect path in the ligand-free form. All these hydration changes induce slight modifications in the protein topology, mostly in presence of enterobactin. The barrel inflation and the plug elongation altogether point to a release of obstruction. The topological analysis gives evidence as to how local modifications of the complex relate to global rearrangements of the water network.

In addition, topological change analysis identifies involved residues, as the widely highlighted Arginine-132 (R132). Biologically interesting, these residues are located in conserved regions (lock region^27^ and channel region^28^) previously reported in relation with transport in FepA (see Table S3).

For future development, it would be interesting to further automate parts of the analysis, in particular for the sake of webserver integration^6^. The computing time, ranging from seconds to minutes on a PC (for ~10^5^ atoms) makes VLDP appropriate for dynamical analysis through series of snapshots, but synchronised update of the topological data along MD trajectories would require deeper modifications of the algorithm. Finally, new developments based on VLDP could improve our understanding of other systems, as hydrolases^29^. The function of these enzymes is regulated by water channels/tunnels and it would be interesting to investigate how VLDP captures this phenomenon.

## Methods

### Simulation protocol

All Molecular Dynamics (MD) simulations were performed using the version 3.1.4 of the GROMACS simulation package (www.gromacs.org) with an extended united atom version of the GROMOS96 force field ffG45a3^30,31^. For consistency, the GROMACS version used is the same as in a previous reference work^18^ for which the system was set up and subsequently extended, although this version is now deprecated. The enterobactin parameters and details on its parametrisation are available for download at the URL http://www.baaden.ibpc.fr/pub/other/ebact.zip. The SPC water model^32^ was employed. The simulation protocol was similar to that used in previous studies of related iron transporters such as FhuA^16,33^. No attempt was made to enforce the iron-enterobactin transport by artificial forces or constraints.

The complete FepA protein (PDBid: 1FEP^23^) was modelled with modeller^34^ to revert selenomethionines to methionines, add missing residues or side-chains and fill out missing parts of incomplete loop regions (L4, L5, L7). The 10 best scoring homology models were analysed and visually inspected (in particular concerning loop conformation) to choose the most adequate one based on several criteria such as RMSD to the original crystal structure template, secondary structure conservation and Modeller energy score. The homology model with both the lowest RMSD and the lowest energy value was then embedded in a pre-equilibrated DMPC bilayer, as described in^35^. Water and ions equivalent to 0.1 M NaCl were added. The equilibration stage of energy minimisation and 0.4 ns of protein restrained dynamics was followed by a 70 ns (for the ligand-free simulation), respectively 86 ns (with ligand), unrestrained MD run in the NPT ensemble. The MD simulation of the complexed form was longer than for the free form in order to capture slow relaxation modes and eventual conformation changes induced by the presence of the ligand. The temperature was maintained at 310 K using a Berendsen thermostat^36^ with *τ* = 1.0 ps. The pressure was maintained at 1 bar by anisotropically coupling *x*, *y* and *z* components to a Berendsen barostat^36^ with *τ* = 1.0 ps and compressibility of 4.5 10^−5^ bar^−1^ in all three dimensions. The time step for integration was 2 fs. Electrostatic interactions were calculated using a cutoff of 18 Å and van der Waals interactions were truncated at 14 Å. The LINCS algorithm^37^ was used to constrain all bond lengths.

Two states of the system were investigated by MD simulation: one with FepA in its ligand-free form, the other in its complexed form including the iron-enterobactin ligand. The iron-enterobactin complex was positioned at the iron site FE1 inferred from an anomalous difference density map with the assistance of the authors of ref.^23^. More precisely, FE1 is the first of two alternative positions found experimentally at fractional coordinates FE1: 0.33120, 0.25000, 0.99038 and FE2: 0.36808, 0.225000, 0.42203 (see ref.^23^). The Fe ion was stabilised by harmonic constraints with six O neighbours in the enterobactin.

The topological analysis was carried out on a set of respectively *n* = 15 MD snapshots for the ligand-free form, 18 for the complexed form, all taken at 5 ns intervals. Most of the results were also checked on ten denser 1 ns separated snapshots covering the final 10 ns, respectively 60–70 ns without ligand and 76–86 ns with ligand.

### Graph Theory Methods

#### Laguerre and Delaunay diagrams

The Laguerre tessellation and its dual, the Delaunay diagram, are partitions of space into polyhedra. Each Laguerre polyhedron^38^ represents and encompasses an atom whereas, in the Delaunay partition, space is divided into tetrahedra whose vertices are atomic positions. Further details about tessellations, applications, and bibliographical references are given in^4,39,40^.

Both tessellations are based on a set of atomic positions and weights. The atomic positions are those of the system comprising the protein, lipids and solvent, but discarding the hydrogen atoms. Any water molecule reduces to a single oxygen atom. In the Laguerre tessellation, all atoms have the same weight, set to zero, except water oxygen which is assigned a special weight *w* to compensate for the absence of hydrogen. The reason why we use the Laguerre weighted tessellation rather than the simple Voronoi one (which is the special case where all weights are equal) is that the geometry, in particular the volumes, are more coherent when changing the description from all-atom to the residue scale, and closer to experimental data^4,38,41^. These weights fine-tune the position of the protein-solvent interface.

The water weight value was determined by minimising the following function:

1 $$f(r)=\sum _{i}^{n}\,{({({\bar{v}}_{i}-{\bar{v}}_{{\rm{ref}}})}^{2}+{({\sigma }_{i}-{\sigma }_{{\rm{ref}}})}^{2})}_{r},$$

where ${\bar{v}}_{i}$ is the average Laguerre volume of water O in snapshot *i*, the average being taken over the water layer at the water-solute interface (solute is either the protein or lipids); the reference ${\bar{v}}_{{\rm{ref}}}$ is the mean Laguerre volume of surface water molecules evaluated in a preliminary computation that included all water hydrogen atoms; *σ*_*i*_,*σ*_ref_ are the corresponding volume standard deviations. The plot of *f* as function of water radius *r* = *w*^1/2^ is provided in Fig. S9. The minimum was computed separately on both sets of snapshots, FepA ligand-free and complexed. In both cases, the minimum is reached at *w* = 1.48 Å^2^, the water weight adopted in all subsequent computations.

#### Contacts, neighbourhood and paths

Two atoms are in contact when their Laguerre polyhedra share a common face. This is equivalent, in the dual Delaunay tessellation, to the presence of a (tetrahedral) edge linking the atoms. In such a situation, the atoms are considered as neighbours. A graph is a set of vertices and a set of edges connecting pairs of vertices. Any tessellation contains a graph, its 1-skeleton, consisting of all the vertices and all the edges of the polyhedra. A path is a chain of vertices *x*_1_, .., *x*_*n*_ in which all pairs of consecutive vertices (*x*_*j*_, *x*_*j*+1_) are neighbours (linked by an edge). Most often, a path of atoms in the Delaunay graph is represented as contiguous Laguerre polyhedra, as the water path in Fig. S3.

#### Connected components in Delaunay and Laguerre diagrams

A graph is connected if it contains a path from any of its vertices to any other vertex. Otherwise, the graph splits up into several connected components, or clusters (maximal connected sub-graphs). To find connected components, VLDP implements a recursive depth first search algorithm^42^. By construction, the complete (full system) Delaunay or Laguerre tessellation defines a connected graph. Separated clusters appear when one considers sub-graphs, for example the sub-graph containing only the water molecules, or the sub-graph defined by a specific list of residues.

To analyse the water network, one first defines the Delaunay sub-graph made of all the water molecules (reduced to water oxygens). Then, in the algorithm, a recursive search lists all connected components. Finally, the main component of the water network is defined by its extensive number of water molecules.

#### Protein surface and genus

The algorithm finding connected components is also used to analyse the surface of the protein represented as a cluster of Laguerre polyhedra. Its surface (or boundary) is the union of all the Laguerre faces at the interface between the protein and outside atoms (solvent or lipids). Now, even if the protein is connected, its surface may not be. So a search of connected components is performed in the Laguerre graph (set of vertices and edges) of the protein surface only.

The list of those connected components contains the outer surface of the protein, separating it from the outside, and smaller closed surfaces bounding water inclusions surrounded by the protein.

The genus is a topological number classifying surfaces. A surface of genus *g* is equivalent to a sphere with *g* handles. *g* is also the maximum number of non-intersecting simple closed curves that can be drawn on the surface without separating it. In the algorithm, the genus *g* is computed from the Euler characteristic *χ* and the Euler-Poincaré formula. In a closed polygonal surface, count the number of vertices *V*, edges *E* and faces *F*. Then

2 $$\chi =2-2g=V-E+F.$$

In VLDP, the genus is evaluated on each connected component of the surface.

In FepA, in addition to the entire protein surface, two sub-domains were considered: the plug domain (residues 1-148) and the peripheral part (loops and *β*-barrel). For each case, the Laguerre tessellation was built on the whole system (protein, lipids and solvent), but only the subset of polyhedra belonging to the specific part was retained to calculate the surface genus.

#### Disjoint Paths

Crossing water paths are found by Dijkstra’s algorithm^9^ providing the shortest path(s). The water network consists of vertices (water O atoms) and Delaunay edges linking those vertices. A sub-domain is delimited by the augmented enclosing sphere, that is, the region inside a ball of radius *R* just large enough to enclose the protein and extended by 1.4 Å, the standard water radius. The searched paths are constrained to start from a water O inside a thin shell in the extra-cellular domain and to end in the periplasm. When the search of disjoint path(s) is iterated, all the water molecules already visited by previous paths are removed from the set of water nodes available for the next paths.

#### Widest Paths

For searching the widest channels, Dijkstra’s algorithm is implemented with a modified potential *φ* inspired from MOLE^10^:

3 $$\phi (x)={(\frac{3\AA }{d(x,{\rm{p}}{\rm{r}}{\rm{o}}{\rm{t}})})}^{6}+m(x)\,{(\frac{3}{{d}_{p}})}^{6},$$

where *d*(*x*, prot) is the distance between the position *x* and the protein, i.e. the distance from *x* to the closest protein atom; *m*(*x*) is the multiplicity of visits while iterating the algorithm; *d*_*p*_ is a fixed parameter setting the overlap penalty. This potential function is evaluated and summed on all the vertices *x* of the path. The algorithm searches the path minimising this global potential. Eq. (3) is set to find a water path as far as possible from amino acids on average. The present algorithm differs from MOLE in two main points. Firstly, path length is not incorporated explicitly. It nevertheless matters because the longer the path, the more terms in the sum. Secondly, the widest path search is performed on the water Delaunay graph but not on the isolated protein Voronoi edges.

The second term on the right in Equation (3) is an overlap penalty imposed when the algorithm is iterated to find several optimal, widest, paths. For each vertex *x*, the multiplicity *m*(*x*) counts the previous paths passing through *x*. Setting *d*_*p*_ to a small value in Eq. (3), typically *d*_*p*_ = 0.1, implies a huge penalty and the resulting optimal paths are strictly disjoint because the iteration stops at a bounded threshold. Setting *d*_*p*_ to a larger value — we used *d*_*p*_ = 1 — softens the penalty and the iterated search provides a bundle of optimal paths mostly, but not completely, separated.

In order to investigate the effect of ligand binding in FepA, the paths were constrained to start from the enterobactin site or envelope. This envelope is the set of residues that remain in contact with enterobactin in all snapshots with ligand. It defines the enterobactin site in FepA even when the enterobactin ligand is absent.

### Software

The topological and geometrical analyses have been carried out by VLDP (Voronoi Laguerre Delaunay Protein)^4–6^, a program developed at the LPTM (Cergy-Pontoise, F). The views of the molecules, polyhedra, channels, etc. were produced using Pymol (version 1.0r2)^43^. Parts of Fig. 1 were produced using HyperBalls^44^. The plots were produced by Gnu R^45^.

## Electronic supplementary material

Supplementary Information
