# Supplementary material for: Analyzing protein topology based on Laguerre tessellation of a pore-traversing water network
Source: Sci Rep. 2018 Sep 10;8:13540. doi: 10.1038/s41598-018-31422-5 (PMC6131185; doi:10.1038/s41598-018-31422-5)
Supplement: Supplementary file 1 — Supplementary Information [file 41598_2018_31422_MOESM1_ESM.pdf]

Supporting Information for:

Analyzing protein topology based on Laguerre tessellation of a pore-traversing water network

J. Esque[1], M. S. P. Sansom, M. Baaden, and C. Oguey[1]

[1]esque@insa-toulouse.fr oguey@ptm.u-cergy.fr

Supporting Figures

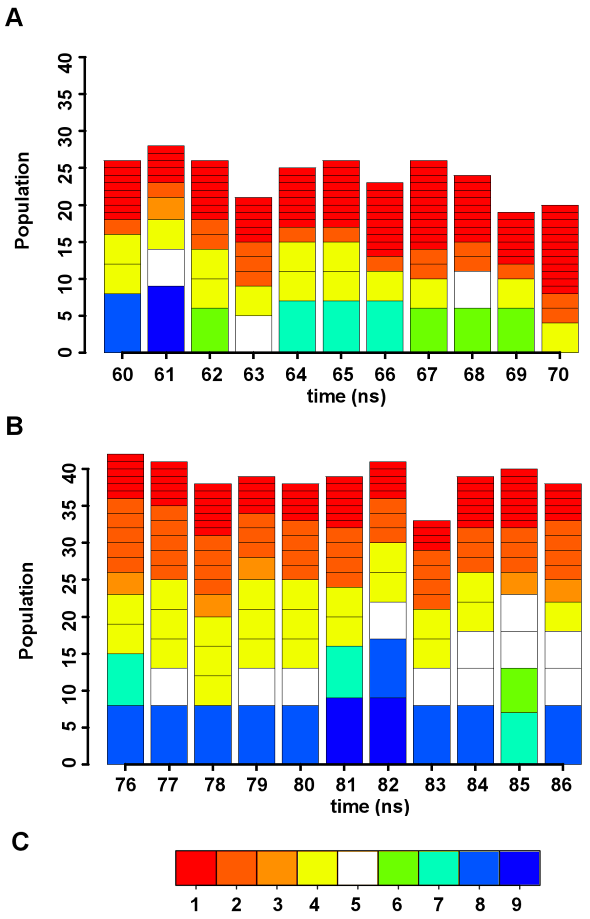

Figure S1. **Population of the isolated water inclusions**. The population (count of water molecules) is plotted for all the indicated snapshots (time) of (**A**) the ligand-free form, (**B**) the complexed form of FepA. Each rectangle represents one inclusion, with population colour code given in (**C**).

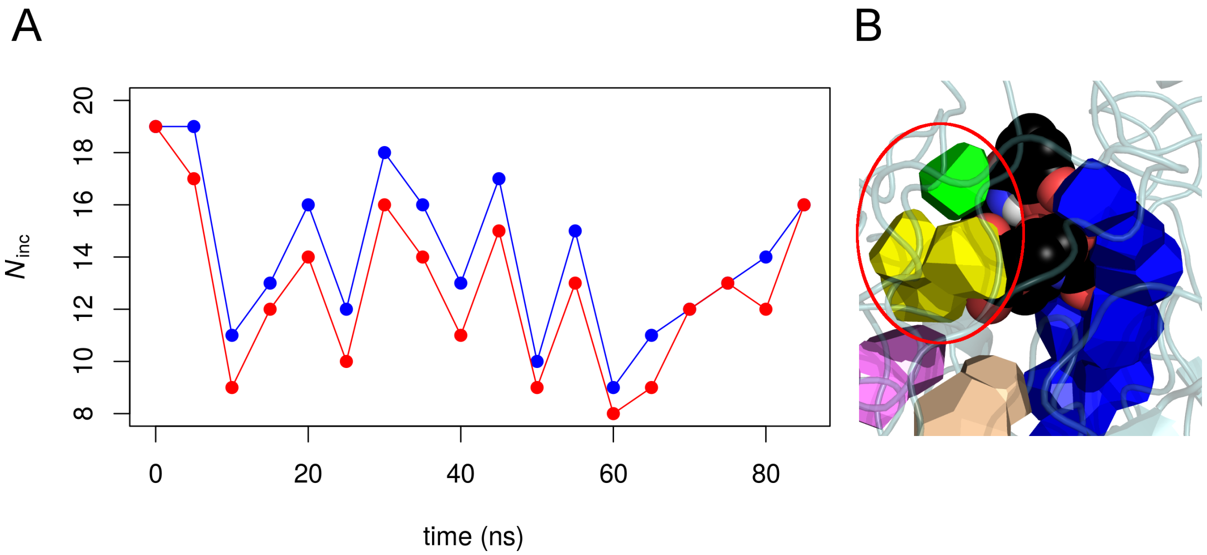

Figure S2. Influence of the enterobactin-iron status on water inclusions. (**A**) The number N_{inc} of isolated water clusters inside FepA complexed with the enterobactin-iron ligand is plotted as a function of MD snapshot time. The enterobactin-iron complex is treated either as protein-like (blue dots) or as solvent (red dots). The blue dots represent the data, N_{inc} complexed, of Table S1. The interpolating lines are visual guides only.
(**B**) This 3D view, extracted from the 80 ns snapshot, demonstrates the change in N_{inc}. The enterobactin-iron complex is shown as van der Waals spheres whereas the protein is in lightteal cartoon. Water molecules are displayed as Laguerre polyhedra. Each connected component has a different colour, randomly chosen, except blue representing the main connected component (as in Figure 2 of the main text). When the ligand is considered as being part of the solvent, it merges the green and yellow water clusters (encircled) with the main component, in blue, thereby decreasing N_{inc} by 2.

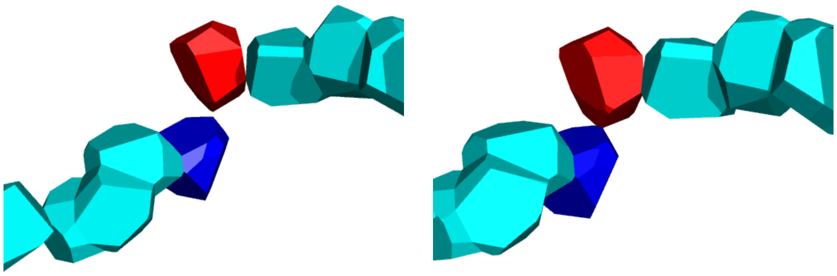

Figure S3. Path disconnection. Example of a string of water polyhedra that is disconnected with standard weight w_{ref} (left) and connects into a path when water has a Laguerre weight 75% larger (right). The images are taken from the ligand-free snapshot at 60 ns.

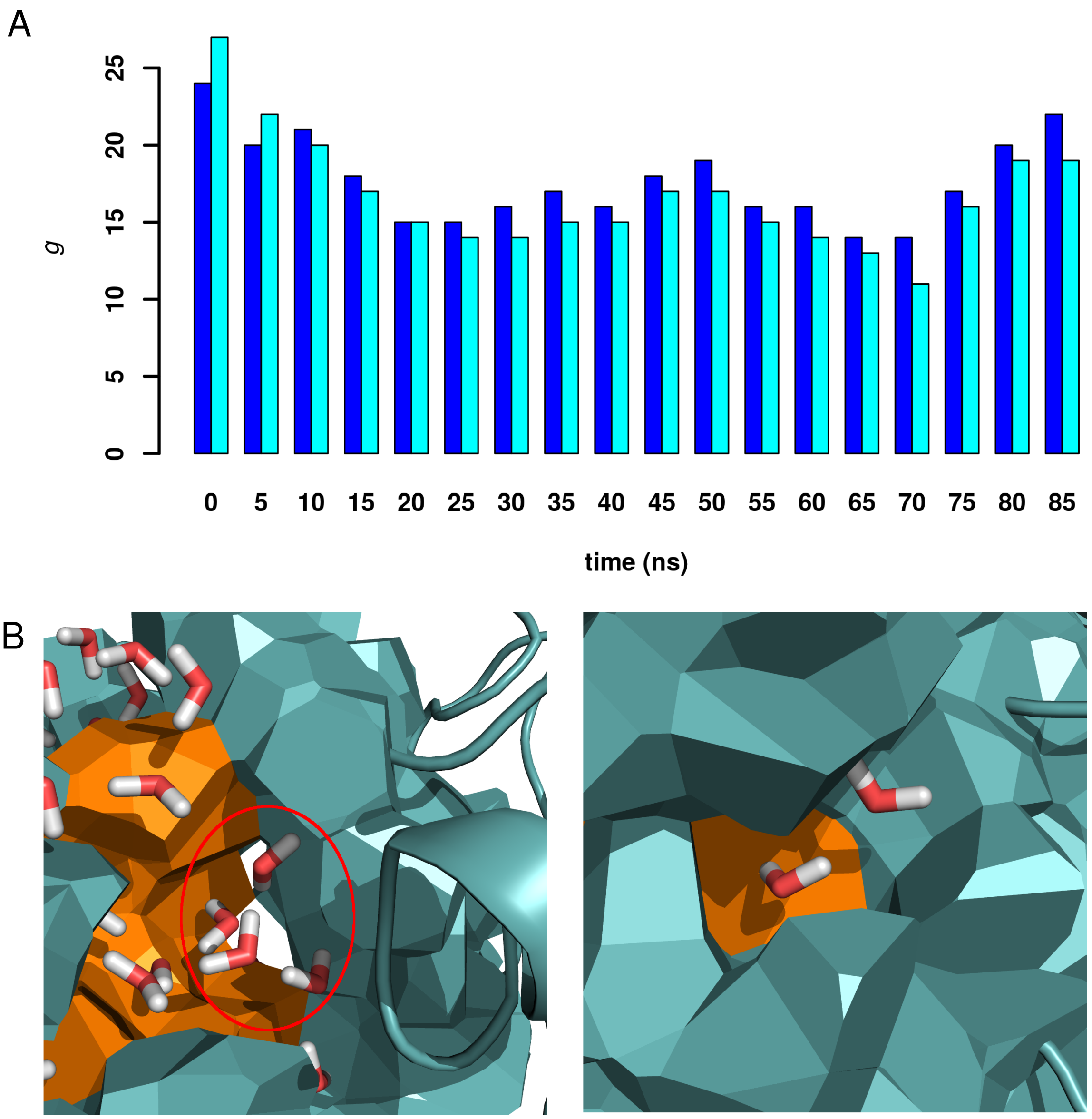

Figure S4. **Influence of the enterobactin-iron status on the protein surface genus.** (**A**) The global genus *g* is plotted as a function of MD snapshot time. The enterobactin-iron complex is treated either as solvent (blue bars) or as protein-like (cyan bars). The blue values are the same as the corresponding ones in Figure 4, complexed case, of the main text. (**B**) The enterobactin-iron is displayed by its orange Laguerre polyhedral surface whereas the FepA protein, coloured lightteal, is represented in a mixed way: cartoon except for the residues in contact with the ligand, which are in atomic Laguerre polyhedra. On the left (at 0 ns), a single opening in the protein alone transforms into two narrower passages in the united enterobactin-iron-protein complex. On the right (at 50 ns), a hole in the protein is shut by the presence of the enterobactin-iron ligand (orange).

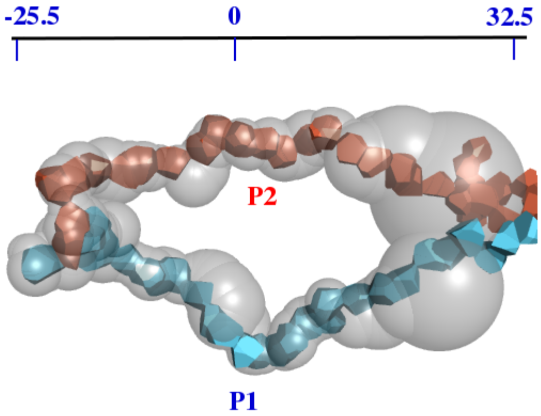

Figure S5. **Two ways of defining optimal water paths.** Method 1: Voronoi algorithm without solvent (inspired from MOLE); paths follow edges of a Voronoi tessellation built on the isolated protein, discarding solvent, lipids and ligand. Each path is a sequence of maximal empty spheres (in grey). Method 2: Laguerre tessellation with solvent; each path is a string of contiguous water Laguerre polyhedra (coloured). In both cases, the optimal paths minimise a potential function penalising short distance with the protein. The comparison is carried out on two paths, P1 and P2, in the complexed form of FepA at 86 ns.

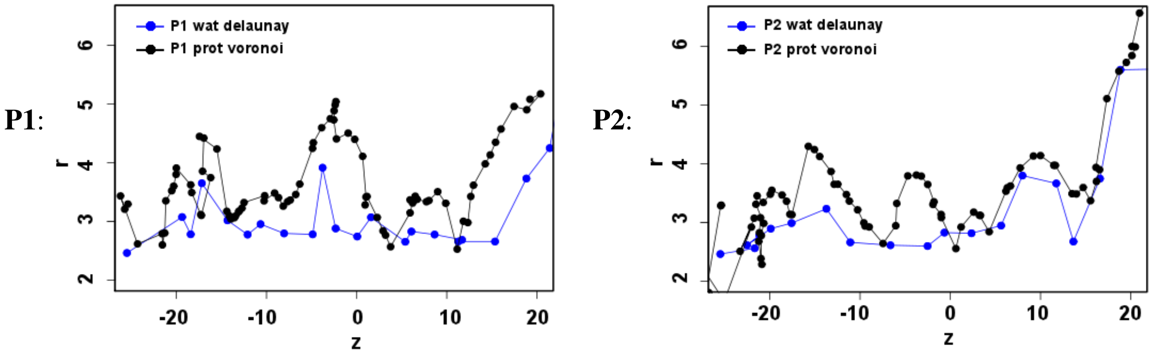

Figure S6. **Channel radius as a function of *z*.** The *z* axis is the protein axis, perpendicular to the membrane. The radius is plotted following a water path (method 2, in blue) or a path in the Laguerre graph of the protein only (method 1, in black), for both paths P1, P2 shown in the previous Figure S5.

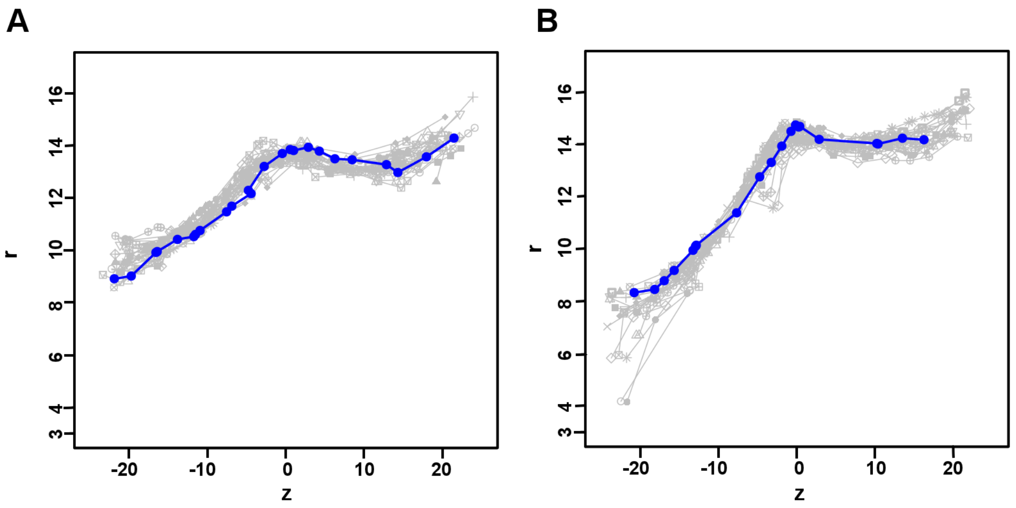

Figure S7. Internal barrel radius as function of z, for FepA apo (A) and holo (B). The internal radius of the barrel was evaluated by a Voronoi method similar to MOLE. Only a peripheral slice of the protein was kept (delimited by $z_{\min}$ and $z_{\max}$ of CA choline headgroup atoms of DMPC lipids); water and the plug domain were removed.The origin $z = 0$ corresponds to the geometrical centre of the peripheral part. Each line represents a snapshot in the last 20 ns (50 to 70 ns for apo, 66 to 86 ns for holo); the blue line highlights the last snapshot.

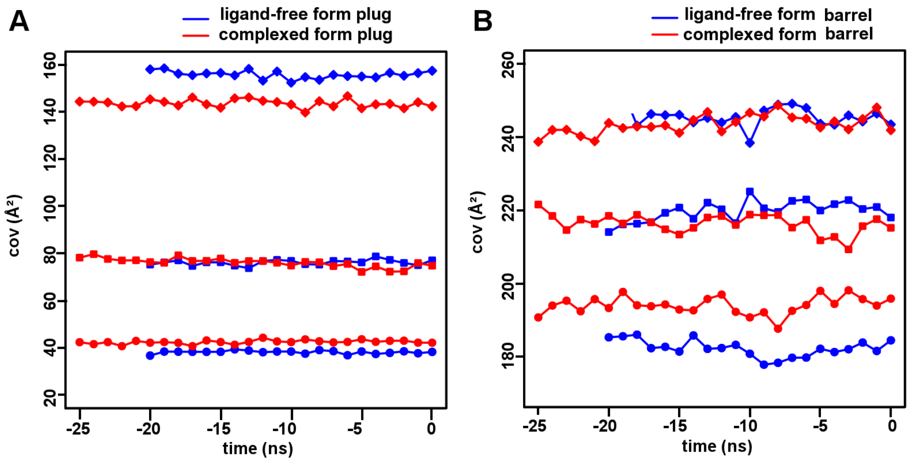

Figure S8. Covariance eigenvalues of the plug (A) and barrel (B). The position-position covariance matrix, related to the matrix of inertia I by $\text{cov} = R^2 - I$ (R is the rms or gyration radius), was evaluated for both the plug domain and the barrel. Here the barrel is the part of the periphery (FepA without plug) delimited by $z_{\min} < z < z_{\max}$ where $z_{\min}$, $z_{\max}$ are the top and bottom of the plug domain. Time is indicated relative to the end of the simulation. In order of increasing eigenvalues, the corresponding principal directions are approximately $\hat{x}, \hat{y}, \hat{z}$ for plug, $\hat{x}, \hat{y} - \hat{z}, \hat{y} + \hat{z}$ for barrel, without any significant change of orientation between the ligand-free (blue lines) and complexed (red lines) forms of FepA.

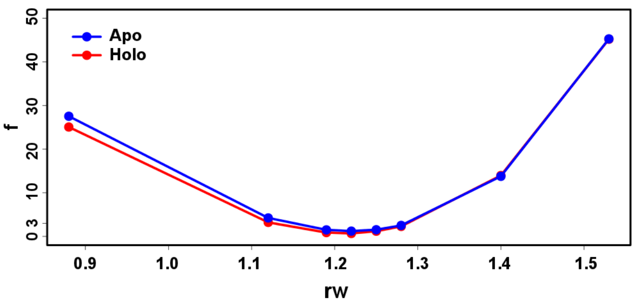

Figure S9. **Cost function f as a function of water radius r.** The cost function is plotted for the free (red) and the complexed (blue) forms of FepA. The water weight w chosen for this study is the square of the minimising radius $r_w = 1.22$ Å.

Supporting Tables

Table S1. **Water inclusions in FepA.** For each snapshot (ligand-free: $0 \rightarrow 70$ ns, complexed: $0 \rightarrow 85$ ns), N_{inc} is the number of inclusions trapped in protein environment. The population, counting water molecules in each inclusion, is reported by the average $\langle\mathrm{pop}\rangle$ and standard deviation σ over the N_{inc} inclusions. The maximal population recorded is 16 molecules for the free form, 15 for the complexed form. The bottom line indicates time averages and rms taken over the 11 snapshots closely sampled in the last 10 ns (see Figure S3 for details).

ligand-free				complexed		
N_{inc}	$\langle\mathrm{pop}\rangle$	σ	time(ns)	N_{inc}	$\langle\mathrm{pop}\rangle$	σ
14	1.4	0.6	0	19	1.8	1.1
8	1.3	0.5	5	19	1.7	1.8
11	1.2	0.4	10	11	2.7	4.2
13	1.2	0.4	15	13	2.0	1.9
15	1.9	1.5	20	16	2.1	1.8
11	1.2	0.4	25	12	1.8	1.1
12	1.4	0.9	30	18	1.7	1.1
13	1.5	1.0	35	16	1.6	1.1
11	1.6	1.5	40	13	2.5	2.4
16	2.4	3.8	45	17	2.3	1.6
12	2.9	4.5	50	10	2.0	1.9
14	3.0	3.8	55	15	2.4	1.8
12	2.2	2.2	60	9	1.9	1.3
13	2.0	1.9	65	11	3.1	2.1
15	1.3	0.8	70	12	2.4	2.1
			75	13	3.2	2.6
			80	14	2.7	2.0
			85	16	2.5	2.1
12.5	1.9	1.70	last 10 ns	14.6	2.7	2.1

Table S2. Stability of disjoint paths as a function of water weights. The water weight is the reference $w_{\text{ref}} = 1.48$ Å^2 times a factor a given in the top row. Each coloured line represents one path.

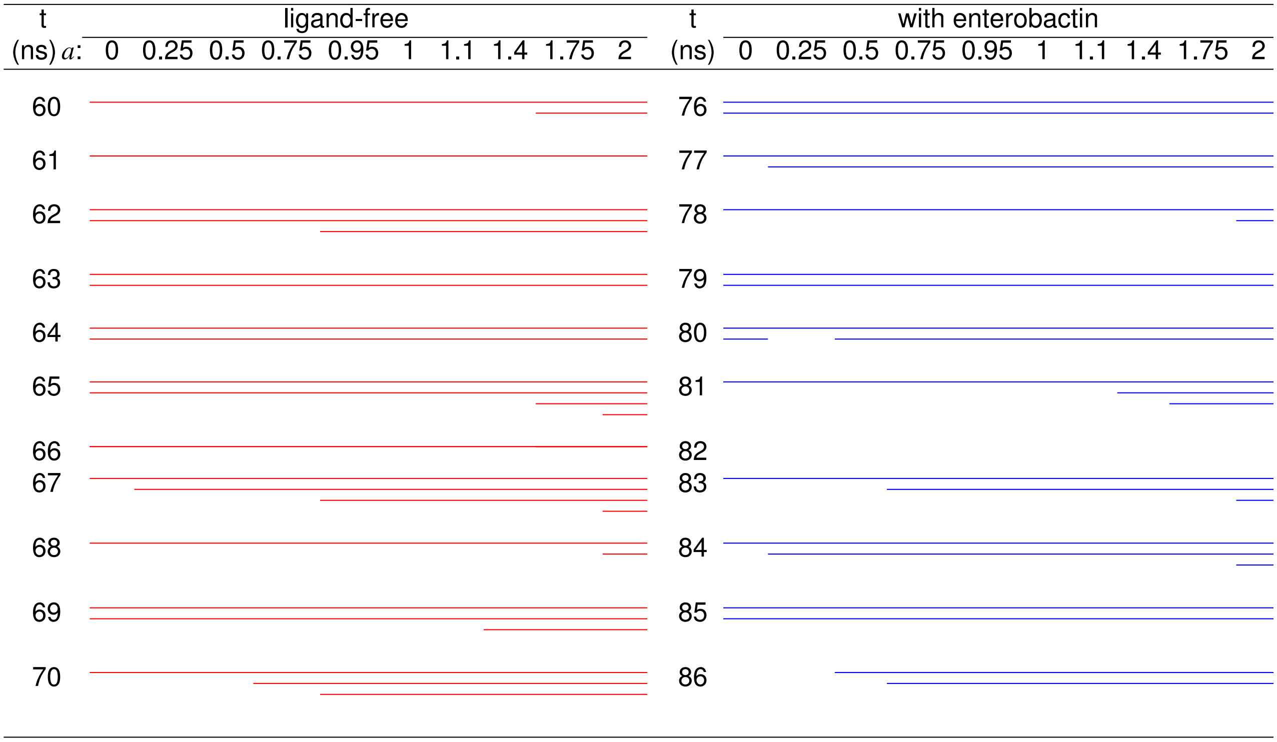

Table S3. Residues bordering main water channels in apo and holo during the last 10 ns. The table lists the residues found bordering in both ligand-free and complexed forms. The colour code is the same as in Figure 8 in the Main Text. Residue labels follow the original pdb file. The conserved residues (Chakraborty, 2003; Lopez, 2007) are surrounded by a square box.

Blue channel:

D12 T13 V16 A19 E20 L23 Q24 D34 R66 I86 D87 G88 K89 P90 S92 S93
N95 S96 R98 G100 E104 D106 R108 W113 V114 M118 E120 R121 E123 R132 Y133 N143
I145 T146 K148 G149 S150 G151 E152 Y192 E226 D232 N234 V236 R238 P243 L244 S246
E248 E250 Q256 N258 Y260 G262 Y272 N282 L284 R286 N288 T292 W293 N294 T302 S304
W306 Q308 E310 T312 N314 R316 E319 I340 D344 E350 Q372 D376 S378 D455 Y472 L480
S482 K517 D519 E576 E617

Green channel:

V28 T30 I31 T32 E35 N39 A42 K47 I48 R50 T51 R66 R121 E152 N162 R225
P243 L244 E331 K517 D519 L522 N571 P573 E576 M579 T581 N583 T610 S612 Q614 E617
Q622 T624 Y628 D676 E711 G713 T715
